# Effect of Non-alcoholic Fatty Liver Disease on Transaminase Levels and Transient Elastography in Patients with Chronic Hepatitis B

**DOI:** 10.7759/cureus.5995

**Published:** 2019-10-25

**Authors:** Asim Sharif, Zaigham Abbas, Samiuddin Ahmed, Shoukat Ali Samjo, Khurram Baqai

**Affiliations:** 1 Gastroenterology, Ziauddin University Hospital, Karachi, PAK; 2 Gastroenterology and Hepatology, Ziauddin University Hospital, Karachi, PAK; 3 Internal Medicine, Ziauddin University Hospital, Karachi, PAK; 4 Hepatology, Ziauddin University Hospital, Karachi, PAK

**Keywords:** non-alcoholic fatty liver disease, chronic hepatitis b, alanine transaminase, transient elastography

## Abstract

Objective

To investigate the effects of non-alcoholic fatty liver disease on aminotransferase (ALT) levels and transient elastography in patients with chronic hepatitis B (CHB).

Methods

A cross-sectional study of 230 patients with CHB and ALT levels up to two times the upper limits of normal, of one-year duration, from June 2018 to May 2019. The demographic, clinical, and laboratory characteristics of each patient were collected. Transient elastography was performed to evaluate controlled attenuation parameter (CAP or steatosis) and liver stiffness (fibrosis).

Results

A total of 161 (70%) patients were overweight, with over two-thirds (166; 72.2%) having elevated ALT >35 U/L. Three-fourths of the patients (178; 77.4%) had a hepatitis B virus (HBV) deoxyribonucleic (DNA) level of less than 2000 IU/ml. Steatosis was detected in 166 (72.2%) patients while fibrosis of F2 or more in 88 (38.3%). Multivariate regression analysis showed that weight, homeostatic model assessment of insulin resistance (HOMA-IR), and elevated ALT levels of more than 35 were independently associated with higher CAP values (p= 0.019, 0.001, and 0.004, respectively). Age, insulin levels, and platelet counts were independently associated with liver elasticity (p=0.00, 0.002, and 0.028, respectively). HBV DNA levels did not show any significant association with CAP score, liver stiffness, and HOMA-IR or ALT level. Among those with an elevated ALT of 35 or above (n=166), 124 patients had HBV DNA levels less than 2000 IU/ml. Out of these, 97 (78.2%) patients had steatosis and 51 (41.1%) had F2 or more fibrosis.

Conclusion

A significant number of patients with CHB with mildly elevated ALT levels are overweight, have significant steatosis and fibrosis, but low HBV DNA levels. This aspect is important while making decisions regarding hepatitis B treatment.

## Introduction

Chronic hepatitis B (CHB) and nonalcoholic fatty liver disease (NAFLD) are two of the most prevalent liver diseases worldwide. About 248 million patients worldwide have CHB [1]. It is estimated that 25%-30% of CHB patients have a coexisting NAFLD [2-3]. Liver biopsy remains the gold standard to diagnose NAFLD, but it is invasive and should be considered in patients with NAFLD who are at increased risk of having steatohepatitis and/or advanced fibrosis [4]. Consequently, more attention is being given to non-invasive imaging studies and markers for the assessment of NAFLD, including ultrasound, transient elastography, and so on.

Vibration-controlled transient elastography is a noninvasive technique to assess hepatic fibrosis and steatosis, and it has been evaluated in patients with chronic hepatitis B and C and NAFLD [5]. Transient elastography utilizes proprietary algorithms based on the ultrasonic attenuation coefficient of vibration-controlled transient elastography, and the liver stiffness measurement (LSM) and CAP are calculated from the returning shear wave velocities, which correlate with hepatic fibrosis and steatosis, respectively [6].

There are patients who have a concurrent CHB virus infection and NAFLD, which can cause a clinical dilemma regarding different clinical management for these two conditions, as the association between chronic hepatitis B virus (HBV) infection and fatty liver is incongruous. The aim of this study is to find out the role of NAFLD and its effect on transaminase levels and elasticity in chronic hepatitis B patients.

## Materials and methods

In this cross-sectional study, subjects were recruited through a non-probability consecutive sampling technique. The duration of the study was one year, from June 2018 to May 2019. The sample size was calculated as Huanhuan Yang et al. did [7]. Adult patients of both genders with chronic hepatitis B, age between 18 and 70 years, with positive hepatitis B surface antigen (HBsAg) documented for at least six months and transaminases of up to two times the upper limit of normal, were recruited. Those with autoimmune liver disease, drug-induced liver injury, alcoholic fatty liver disease, viral liver disease, and contraindications to transient elastography examination (e.g., ascites, non-healing wounds in the upper-right quadrant of the abdomen, pregnancy, etc.) or unpredictable CAP measurements (e.g., success rate less than 60% or interquartile range (IQR) <30) [8] were excluded from the study. Those who fulfilled the criteria for antiviral treatment were treated according to standard international guidelines.

**Figure 1:**
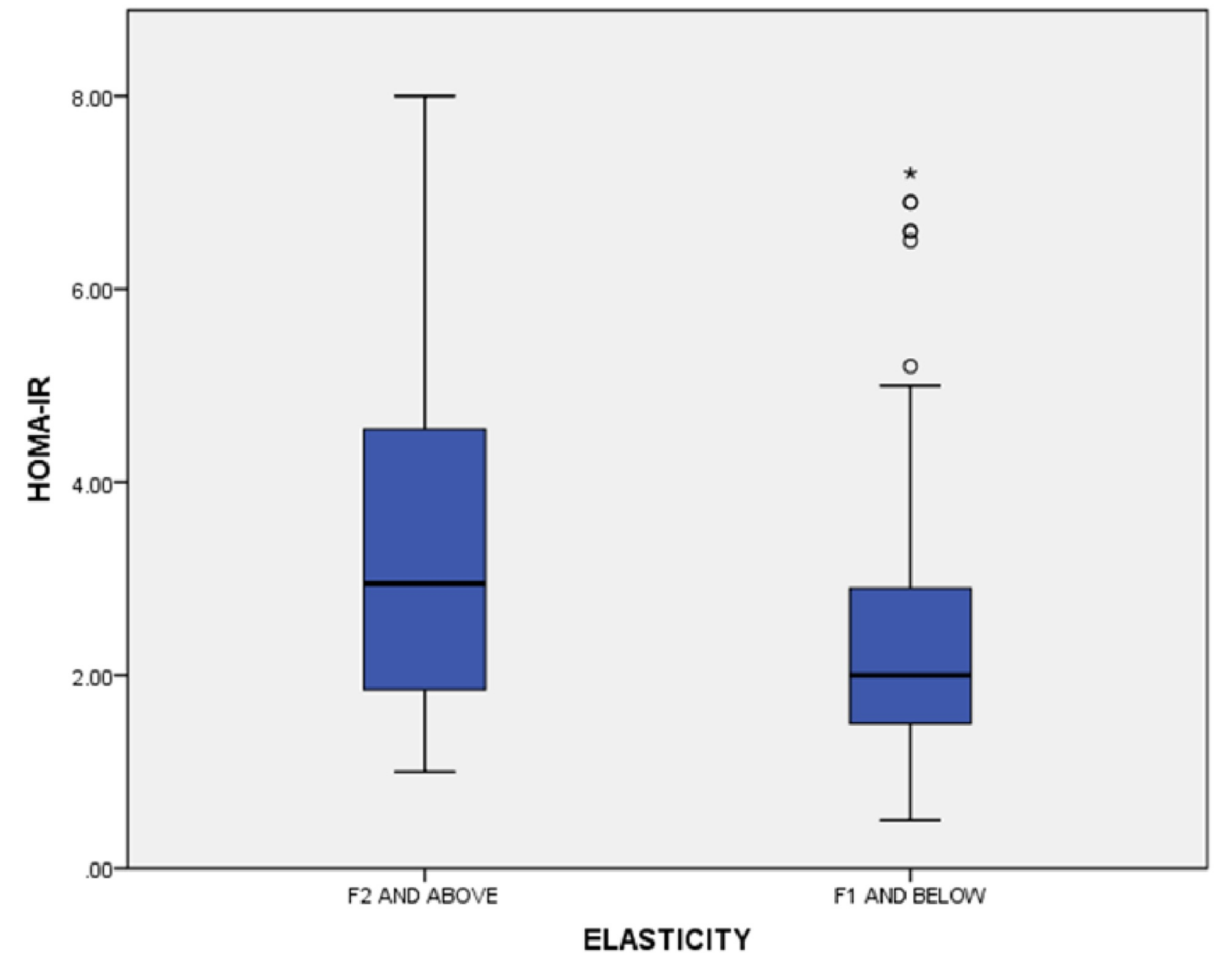
Liver elasticity versus HOMA-IR HOMA-IR: homeostatic model assessment insulin resistance

Written informed consent was obtained from all participants and approval was acquired from the ethical review committee. The demographic, clinical, and laboratory characteristics of each patient were collected, including gender, age, height, weight, body mass index (BMI), medical history, alanine transaminase (ALT), aspartate aminotransferase (AST), gamma-glutamyl transferase (GGT), uric acid, serum creatinine (CR), plasma total cholesterol (TC), triglyceride (TG), high-density lipoprotein (HDL), low-density lipoprotein (LDL), fasting plasma glucose (FPG), fasting insulin, platelet (PLT), hepatitis B virus DNA by quantitative polymerase chain reaction (PCR), and hepatitis B e antigen (HBeAg). Insulin resistance was calculated by the homeostatic model assessment of insulin resistance (HOMA IR) model (Figure 1). Transient elastography of the liver was performed. All information was contained in a predesigned proforma. ALT cut-off levels were according to the American Association for the Study of Liver Diseases (AASLD) 2018 guidelines for HBV: ALT ≥ 25 IU/L for women and ≥ 35 IU/L for men.

The Fibrotouch® Diagnostic system (HISKY Medical Technologies, Beijing, China) was used to capture both the ultrasound-controlled attenuation parameter (UAP/CAP) for steatosis and the liver stiffness measurement (LSM) values for elasticity and fibrosis simultaneously. Attenuation parameter values and LSM values were measured in units of decibels per meter (dB/m) and kilopascal (kPa), ranging from 100 to 400 dB/m and 2.5 to 75 kPa, respectively.

The estimated cutoff taken for F2 was 7.9 (range, 6.1-11.8) kPa, F38.8 (range, 8.1-9.7) kPa, and for F4, it was 11.7 (range, 7.3-17.5) kPa [9]. CAP values for the estimation of steatosis were taken as S0 or normal liver with CAP <238 db/m, an indicator <10% liver fat, S1 or mild fatty liver (11%-33% fatty liver) with CAP >238 db/m but <259 db/m, S2 or moderate fatty liver (34%~66%) with CAP >259 db/m but <292 db/m, S3 or severe fatty liver (> 67%) with CAP >292 db/m [10].

The data were analyzed by Statistical Packages for Social Science version 21 (SPSS Inc., Chicago, IL). Mean and standard deviation were computed for continuous variables and analysis was performed by the Student t-test or Mann Whitney test. Frequency and percentage were computed for categorical observations and analysis was done by using Chi-square, or Fisher’s exact tests, as appropriate. The correlation was assessed by the Pearson correlation coefficient. A step-wise linear regression analysis was performed to identify independent variables associated with elasticity and steatosis. A two-sided p-value of less than 0.05 was considered statistically significant.

## Results

Included in this study were 230 patients; 203 (88.3%) were males and 27 (11.7%) were females. Thirty-three (14.3%) were diabetic and 24 (10.4%) were hypertensive; none had a history of ischemic heart disease. The baseline features of the study subjects are shown in Table 1. Among the study participants, 29 (12.6%) were HBeAg positive. Fifty-two (22.6%) had HBV DNA levels of 2000 or more, 142 (61.7%) had F1 fibrosis or below, 16 (7%) had F2 fibrosis, 33 (14.3%) had F3 fibrosis, and 39 (17%) had F4 fibrosis as determined by transient elastography. The number of patients who were overweight (BMI 25 or more) was 161 (70%). Patients with elevated ALT of more than 35 were 166 (72.2%). Among 33 diabetics, 25 (76%) had elasticity values of F2 or more (p-value=0.00). One-hundred six (46.1%) had HOMA-IR of 2 and more. One-hundred fifty (65.2%) had CAP values of S2 or more. Among 178 subjects with HBV DNA of less than 2000, 15 had reactive HBeAg while 14/52 subjects with HBV DNA of more than 2000 had reactive HBeAg (p-value = 0.001). The distribution of HBeAg was the same across different CAP categories (p-value=0.53). Among those with elevated ALT 35 or above, 124/166 patients had HBV DNA levels of less than 2000. Out of these, 97 (78.2%) patients had steatosis and 51 (41.1%) had F2 or more fibrosis.

**Table 1:** Baseline characteristics of study subjects BMI, body mass index; Hb, hemoglobin; TLC, total leukocyte count; ALT, alanine aminotransferase; AST, aspartate aminotransferase; ALT, alanine aminotransferase; AST, aspartate aminotransferase; GGT, gamma-glutamyltransferase; ALP, alkaline phosphatase; CHOL, cholesterol; TG, triglyceride; HDL, high-density lipoprotein; LDL, low-density lipoprotein; VLDL, very low density lipoprotein; UA, uric acid; HOMA-IR, homeostasis model assessment of insulin resistance

VARIABLE (N=230)	MEAN ± SD	Range
AGE (years)	36.8±10.84	18-70
HEIGHT (cm)	171.8±9.68	134-192
WEIGHT (kg)	80.8±16.1	40-123
BMI (kg/m^2^) BMI≥25 (161/230, 70.0%)	27.2±4.8	14.2-40.6
Waist (cm)	94.6±11.1	65-130
HB (g/dl)	14.01±1.6	8.3-18.1
TLC (x10^9^/liter).	7.07±1.8	2.3-13
PLATELETS (x10^9^/liter).	245±67.6	34-465
T.BILIRUBIN (mg/dl)	0.7±0.46	0.2-3.1
ALT(U/L) ALT≥35 (N=166/230, 72.2%)	51.9±30.3	10-211
AST (U/L)	35.6±16.1	12-115
GGT (U/L)	39.5±32.7	9-258
ALP	87.8±39.6	23-299
CR (mg/dl)	0.7±0.39	0.3-5.7
CHOLESTROL (mg/dl)	173.7±35.1	73-300
TRIGLYCERIDE (mg/dl)	158.1±83.8	46-689
HDL (mg/dl)	41.1±9.5	21-94
LDL (mg/dl)	99.5±31.1	32-210
VLDL (mg/dl)	20.8±14.05	3-80
URIC ACID (mg/dl)	4.8±1.3	2-9.2
INSULIN (µIU/mL)	11.7±6.5	2.05-36
HOMA-IR	2.7±1.5	0.5-8
CAP (dB/m) CAP ≥2(143/230,62.2%)	267.43±57.2	110-400
ELASTICITY (kPa) ELASTICITY ≥F2 (87/230, 37.8%)	9.2±7.5	4.1-75

HBV DNA levels did not show any correlation with liver stiffness (p-value=0.20), CAP score (p-value=o.35), HOMA-IR (p-value=0.23), or ALT levels (p-value=0.63). Liver steatosis, as determined by the attenuation parameter, strongly correlated with age, BMI, weight and waist, platelets, ALT (OR=1.72, 95% CI: 1.21-2.45), AST, GGT, cholesterol, LDL, uric acid, insulin, and HOMA-IR, as shown in Table 1. Further, multivariate regression analysis showed that weight (β =0.25, t value=2.3, p-value=0.019), HOMA-IR (β =0.45, t value=2.47, p-value=0.014), and elevated ALT levels of more than 35 (β =0.18, t value=2.93, p-value=0.004) were independently associated with controlled attenuation parameter values. Univariate analysis revealed a significant correlation of elasticity with age, BMI, waist, platelet count, AST, GGT, ALP, CAP score, insulin, and HOMA-IR, as shown in Table 2. Multivariate linear regression analysis showed that increasing insulin levels (β =0.2, t value=2.8, p-value=0.005), decreasing platelet count (β=-0.15, t value=-2.23, p-value=0.028) and rising age (β =0.2, t value=3.5, p-value=0.00) were independently associated with liver elasticity (Table 3).

**Table 2:** Factors associated with increased attenuation index (steatosis) Data are shown as numbers (percentage), or mean ± standard error of mean. BMI, body mass index; Hb, hemoglobin; TLC, total leukocyte count; ALT, alanine aminotransferase; AST, aspartate aminotransferase; ALT, alanine aminotransferase; AST, aspartate aminotransferase; GGT, gamma-glutamyltransferase; ALP, alkaline phosphatase; CHOL, cholesterol; TG, triglyceride; HDL, high-density lipoprotein; LDL, low-density lipoprotein; VLDL, very low density lipoprotein; UA, uric acid; HOMA-IR, homeostasis model assessment of insulin resistance; HBV, hepatitis B virus; DNA, deoxyribonucleic acid

Variable	Steatosis present N=161, 70%	Steatosis absent N=69, 30%	P-value
AGE (years )	38.08±10.30	33.8±11.5	0.00
BMI (kg/m^2^)	28.3±4.4	24.9±4.7	0.00
WAIST (cm)	96.7±11.0	89.8±10.0	0.00
WEIGHT (kg)	84.0±15.7	73.2±14.4	0.00
HB (g/dl)	13.9±1.62	14.1±1.81	0.52
TLC (x10^9^/liter).	7.18±1.80	6.8±1.9	0.20
PLATELETS (x10^9^/liter).	253.9±65.5	226.1±69.01	0.005
ALT (u/l)	52.39±27.5	50.9±36.2	0.003
GGT (u/l)	43.2±36.2	30.7±20.0	0.001
AST (u/l)	35.6±14.8	35.6±19.07	0.041
ALP (u/l)	87.9±39.6	87.4±39.9	0.92
ALBUMIN (g/dl)	4.03±0.23	4.00±0.28	0.35
CR (mg/dl)	0.74±0.44	0.72±0.23	0.75
CHOL (mg/dl)	177.9.2±34.1	163.7±35.7	0.005
TG (mg/dl)	160.9±80.04	151.84±92.2	0.45
HDL (mg/dl)	40.57±8.78	42.4±11.04	0.178
LDL (mg/dl)	102.2±31.3	93.4±30.1	0.049
VLDL (mg/dl)	21.5±14.4	18.97±12.93	0.176
UA (mg/dl)	5.12±1.35	4.28±1.14	0.000
ELASTICITY (kpa)	9.34±6.17	9.01±10.06	0.061
INSULIN (µIU/mL)	12.98±6.67	9.02±5.59	0.000
HOMA-IR	3.02±1.61	2.03±1.24	0.000
HBV DNA (IU/ml)	4502857.44±29470083.3	8085400.29±34973918.8	0.607

**Table 3:** Factors associated with increased elasticity Data are shown as number, percent, and mean ± standard error of mean. ALT, alanine aminotransferase; AST, aspartate aminotransferase; BMI, body mass index; ALT, alanine aminotransferase; AST, aspartate aminotransferase;GGT, gamma-glutamyltransferase; ALP, alkaline phosphatase; CHOL, cholesterol; TG, triglycerides; HDL, high-density lipoprotein; HOMA-IR, homeostasis model assessment of insulin resistance; LDL, low-density lipoprotein; VLDL, very low density lipoprotein; HBV: hepatitis B virus; DNA: deoxyribonucleic acid

Variable	F 2 and above N=88, 38.26%	F 1 and below N=142, 61.73%	P-value
AGE (years)	8839.7 ±10.8	34.9 ±10.6	0.001
BMI (Kg/m2)	29.4 ±6.7	26.4 ±4.2	0.000
WAIST (cm)	96.2 ±16.2	92.8 ±9.6	0.004
WEIGHT (Kg)	84.9±18.4	78.2±13.9	0.1
HB (g/dl)	13.7±1.64	14.1±1.69	0.15
TLC (x10^9^/ l )	7.06±2.07	7.08±1.73	0.62
PLATELETS (x10^9^/liter).	233.03±65.8	253.4±67.81	0.023
ALT (U/L)	54.7 ±31.2	50.2 ±29.7	0.114
GGT(U/L)	48.2 ±40.8	34.1 ±25.2	0.001
AST (U/L)	38.3 ±17.2	33.9 ±15.2	0.012
ALP (U/L)	94.8 ±42.1	83.4 ±37.4	0.025
ALBUMIN (g/dl)	4.0 ±.25	4.0 ±.24	.249
CR (mg/dl)	0.75 ±.59	0.72 ±.19	0.066
CHOL (mg/dl)	173.3 ±31.8	173.9 ±37.1	0.945
TG (mg/dl)	156.2 ±55.4	159.3 ±97.4	0.103
HDL (mg/dl)	41.5 ±8.6	40.8 ±10.0	0.291
LDL (mg/dl)	101.5 ±31.9	98.5 ±30.7	0.584
VLDL (mg/dl)	19.6 ±11.6	21.5 ±15.3	0.895
UA (mg/dl)	5.0 ±1.4	4.7 ±1.2	0.296
CAP (dB/m)	284.6 ±62.4	256.7 ±51.1	0.000
INSULIN (µIU/mL)	14.3 ±6.6	10.4 ±6.2	0.000
HOMA-IR	3.3 ±1.7	2.3 ±1.3	0.000
HBV DNA (IU/ml)	7877356.65±33707688.8	3983826.67±28727953.8	0.753

## Discussion

There are conflicting studies of hepatitis B associated with NAFLD. Most commonly, it reflects the metabolic profile of the affected individual. In our study, 161 (70%) patients were overweight (BMI 25 or more). Patients with elevated ALT of more than 35 were 166 (72.2%) and 143 (62.2%) had moderate to severe liver steatosis as determined by the controlled attenuation parameter. A population-based Chinese cohort study underscored the importance of metabolic factors as a cause of NAFLD in hepatitis B and excluded any possible relationship between viral factors and NAFLD but observed that in a subgroup of subjects with concurrent type 2 diabetes mellitus, detectable HBV DNA levels were negatively associated with the development of NAFLD (HR, 0.37; 95% CI, 0.14‐0.98) [11]. Peng D, by using liver biopsy as a tool, concluded that apart from metabolic factors, the hepatitis B virus indirectly facilitates the development of steatosis, as there existed a correlation between ALT levels, HBV DNA, and steatosis [12]. A large Chinese cohort analysis also alluded to the significance of metabolic factors for the development of steatosis but incidentally found that the majority of the study subjects were HBeAg positive with no clear association with fatty liver [13]. Our study did not find any significant association between liver steatosis and HBV viral load.

Ultrasound for the assessment of fatty liver has significant intra- and inter-observer variability [14]. CAP can be used in place of liver biopsy along with the metabolic panel to quantify liver fat. A meta-analysis connoted that CAP showed a significant correlation with steatosis on biopsy and BMI; BMI being a confounding factor [15]. One study implied that inflammation or fibrosis did not affect CAP values [16]. Another study revealed that severe steatosis, defined as a CAP value of more than 280 dB/m, is associated with severe fibrosis in patients with chronic hepatitis B [17]. Liver stiffness measurement seems superior to the biomarker panel for liver stiffness evaluation but augmented inflammation evidently, as raised ALT may lead to falsely elevated stiffness values grading fibrosis [18]. Our multivariate analysis showed that weight, HOMA-IR, elevated ALT levels of more than 35, and significant fibrosis of F2 or more were independently associated with CAP. NAFLD represents a common cause of mildly elevated ALT levels [19]. We wanted to see the impact of NAFLD on chronic hepatitis B and only those patients were included who had elevated ALT up to two times the upper limit of normal. Our analysis revealed that ALT strongly correlated with steatosis of CAP 2 or more (p-value=0.003, OR=1.72, 95% CI: 1.21-2.45). Multivariate analysis also showed ALT of 35 or more as an independent factor associated with steatosis.

A study suggested the potential suppressing effects of steatosis on viral replication [20]. Analogously, a large Korean cohort study implied that the presence of HBsAg was associated with a decreased risk of developing non-alcoholic fatty liver [21]. The inverse correlation of viral load with controlled attenuation parameter (Pearson correlation -0.114) could not reach a statistically significant value (p=0.086) in our analysis.

Physical parameters need to be inculcated in the assessment of NAFLD-hepatitis B, as increased BMI has been seen to delay improvement in fibrosis stage in chronic hepatitis B patients under treatment [22]. Concomitant metabolic syndrome also augments the progression of liver fibrosis irrespective of viral infective markers [23]. Moreover, fatty liver in chronic hepatitis B patients is an independent risk factor for hepatoma development, increasing the risk by 7.3 fold as revealed in a retrospective cohort [24]. Along with the development of hepatoma, liver steatosis is an important factor for mortality in chronic hepatitis B [25]. We found a strong association of liver steatosis and elasticity with important factors related to metabolic syndrome.

Insulin resistance is one of the key pathophysiological mechanisms in metabolic syndrome. An Indian study concluded that insulin resistance, as calculated by HOMA IR, is found to be significantly higher among hepatitis B subjects with fatty liver, also showing serum triglyceride levels as an independent factor for steatosis development [26]. Similarly, a large Korean study demonstrated that insulin resistance is associated with chronic hepatitis B virus infection as detected by HOMA-IR and other methods, without any previous history of diabetes mellitus [27]. Wang CC et al. concluded that chronic HBV infection did not seem to be associated with insulin resistance or hepatic steatosis in HBV carriers [28]. Moreover, insulin levels happen to be associated with advanced liver fibrosis [29]. Our paper analyzed the effects of serum insulin and HOMA-IR on liver steatosis, HBV DNA levels, and liver elasticity. Both parameters showed a statistically significant association with elasticity and CAP values but no relationship was found with the HBV viral load.

Diabetes and hepatitis B is another avenue for research. A large cohort study elucidated that those patients with chronic hepatitis B who developed diabetes were at increased risk of progression to cirrhosis and associated complications [30]. Subjects with diabetes in our study had relatively higher elasticity values but the effects on other parameters could only be extrapolated with a larger number.

Our study has certain limitations. The impact of NAFLD on the further elevation of ALT beyond two times the upper limit of normal was not addressed in our study. Moreover, liver biopsy was not done in our patients, which is considered the gold standard for the grading and staging of inflammation, fibrosis, and steatosis. Another limitation of our study is its cross-sectional nature. We also did not extrapolate the effects of antiviral treatment on steatosis.

## Conclusions

We addressed the effect of NAFLD on different physical and biochemical parameters in chronic hepatitis B. As both liver steatosis and hepatitis B are important risk factors for the development of cancer, it is vital to devise a screening strategy for NAFLD in hepatitis B patients in the wake of the rising prevalence of metabolic syndrome and NAFLD.
